# Valsartan/Sacubitril induced isolated angioedema of uvula: A case report

**DOI:** 10.1016/j.heliyon.2024.e39423

**Published:** 2024-10-16

**Authors:** Maisa A. Nazzal, Abbas Iter, Ameed Q. Dawabsheh, Majd A. Bsharat

**Affiliations:** a Department of Infection Control and Prevention, Ibn Sina Specialized Hospital, Jenin, P200, Palestine; b Department of Internal Medicine, Ibn Sina Specialized Hospital, Jenin, P200, Palestine; c Department of Radiology, Ibn Sina Specialized Hospital, Jenin, P200, Palestine

**Keywords:** Angiotensin-converting enzyme inhibitors (ACEI), Angiotensin-receptor blockers (ARBs), Angioedema, Drug-induced adverse effect, Bradykinin level, Palestine, Awareness

## Abstract

**Objective:**

To report a case of drug-induced isolated angioedema secondary to the use of Entresto (Valsartan/Sacubitril).

**Case summary:**

A 75-year-old White man presented with swelling of the uvula with a normal tongue shape and gradual onset of speech difficulty, shortness of breath, and difficulty swallowing after taking his chronic medication Entresto (sacubitril/valsartan). The main possibility considered was uvular angioedema, other potential causes were not identified. The angioedema subsequently resided after discontinuation of the medication and observation. The patient was diagnosed with Quincke's disease, specifically isolated angioedema of the uvula, which was attributed to the use of Entresto (specifically, the valsartan component).

**Discussion:**

Angiotensin-converting enzyme inhibitors (ACEI) are frequently linked to drug-induced angioedema, which is likely attributable to their effects on bradykinin levels. If elevated bradykinin levels are the primary reason behind angioedema owing to ACE inhibitor use, ARBs are thought to cause very few, if any, occurrences of the condition. There have been numerous cases of angioedema that may have been reported by ARBs. The precise mechanism by which each of these classes of medications causes angioedema is uncertain. The expression and activation of AT2 receptors may be induced by rising angiotensin II levels. ARBs have been demonstrated to elevate bradykinin levels in animal models, an effect that is assumed to be attributable to elevated AT2 receptor stimulation. By inhibiting AT1 receptors and elevating angiotensin II levels, ARBs may exacerbate angioedema.

**Conclusion:**

This is one of the first case reports in Palestine of Valsartan/Sacubitril-induced angioedema. This case and the relevant scientific literature are consistent with the hypothesis that ARB causes angioedema. Practitioners should be aware of this potential adverse effect of valsartan although the underlying cause is still not known.

## 1 Introduction

Angioedema, also called Quincke edema, can cause swelling of the oral floor, tongue, lips, and larynx, which, if left untreated, can obstruct the upper airways, and cause severe respiratory distress. It is a condition that may be fatal because it can manifest in multiple body regions [[1], [2], [3], [4]]. An inherited lack of C1 esterase inhibitor, trauma, dietary sensitivity, and medications are a few of the causes of this condition [3].Moreover, angioedema can manifest at different times. Even though angioedema can occur at any point during the duration of treatment, the majority of reported cases were documented within the first 30 days of exposure to Angiotensin-converting enzyme inhibitors (ACEI) [5,6]. Due to the difficulty in determining the connection between the onset of the ACEI therapy and symptoms, delayed angioedema may be linked to inadequate detection [5].

ACEIs are one of the most prominent medications used to treat congestive heart failure, hypertension, and proteinuria in diabetic and non-diabetic nephropathy with enhanced results such as renal and cardiovascular protection in patients with diabetes [7,8].

Despite the fact that ACEIs are well known, side effects are nevertheless possible. An adverse reaction that could be fatal can occur in 0.1 %–0.7 % of people using ACEIs. A common cause of acquired angioedema that accounts for 25–40 % of all cases is ACEI. It is estimated that the overall incidence of angioedema ranges between 0.1 % and 0.5 %. Moreover, in some studies, angioedema as an adverse reaction to ACEI medications is under-recognized, whereas in others, the opposite occurs [6,[9], [10], [11]].

Since the precise mechanism is uncertain, it is believed that ACEI-induced angioedema is predominantly caused by the enzymatic suppression of bradykinin breakdown, despite having potential therapeutic benefits [10]. Many variants of this condition still have not been fully understood regarding pathophysiology, causation, and management [12,13].

Angiotensin-receptor blockers (ARBs), which are not known to impact bradykinin levels, should not have the same effect as ACEI-associated angioedema if the bradykinin increase is the underlying cause. ARB-induced angioedema has been recorded since ARBs first hit the market, and valsartan-induced angioedema was reported in a couple of studies [3,10,14].In addition to the other component of Entresto, sacubitril is a neprilysin inhibitor, which leads to reduced breakdown and increased concentration of endogenous natriuretic peptides as well as increased levels of vasoconstricting hormones such as angiotensin II which leads to increased levels of bradykinin. However, when combined with valsartan, it would block angiotensin II to its receptor, preventing the vasoconstrictive effects and decreasing vascular resistance and blood pressure. This case was reported to assess the probability that Entresto caused the adverse event in our patient and to increase awareness of the pharmacologic and adverse effect profile of these agents.

## 2 Case

A 75-year-old man was brought to the emergency department 5 h after experiencing a gradual onset of speech difficulty, shortness of breath, difficulty swallowing, and noisy breathing. The patient was non-Hispanic white in race and ethnicity. He has a history of smoking and has never taken ACEIs or ARBs before starting Entresto, the patient denied any history of a similar condition. He has a medical history of hypertension for three years, diabetes mellitus for one year, ischemic heart disease for 20 years (with a previous coronary artery bypass graft surgery), and heart failure with an ejection fraction of 40 %, which is being managed with optimal medical therapy including aspirin 100 mg, bisoprolol 5 mg, dapagliflozin 10 mg, atorvastatin 40 mg, Entresto (sacubitril/valsartan) 100 mg, and tamsulosin 0.4 mg. The patient was on Entresto for a total duration of more than two years.

Based on the patient's chronic medications including bisoprolol, aspirin, tamsulosin, dapagliflozin, and atorvastatin. The patient has been on aspirin, bisoprolol, and atorvastatin for a long time and there is no history of any adverse effects including angioedema while taking these medications. No such cases have ever been reported for atorvastatin and it is rare to occur with bisoprolol, tamsulosin, and dapagliflozin. Since the patient did not have any previous history of ACEI angioedema and did not take any angiotensin-II antagonists (ARBs) previously. This increases the possibility of ARB-induced angioedema. Also, the mechanism of action of ARBS does not have a direct effect on bradykinin but still, it is one of the medications that is known to cause angioedema. There is a risk that it could be attributed to sacubitril but sacubitril is not given as monotherapy, it is always combined with other medication, and the patient has not taken ARBs before. Therefore, we assumed it was Entresto-induced angioedema.

**Fig. 1:**
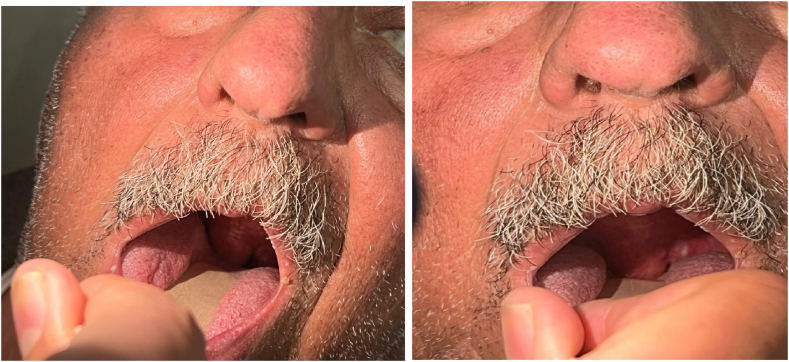
Day two after the event occurred with the patient, swollen uvula with normal tongue shape.

**Fig. 2:**
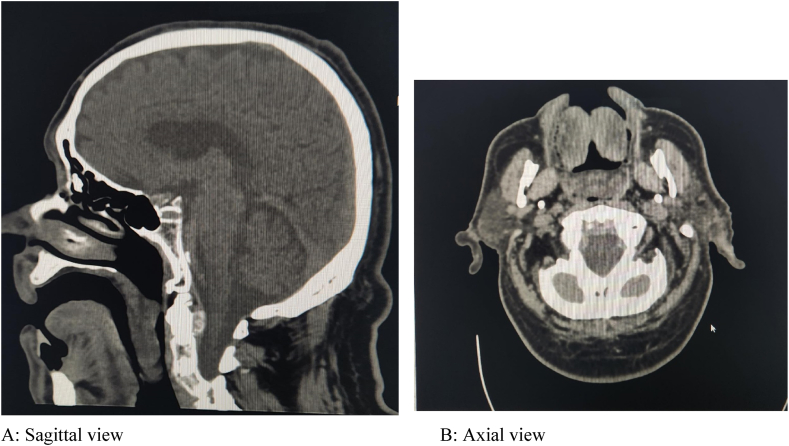
Brain CT scan, A: Sagittal view, B: Axial view: A pedunculated soft tissue mass measuring approximately 5 × 2 cm in the oropharynx, attached to the soft palate.

The patient denies any history of headache, limb weakness, mouth deviation, orthopnea, chest pain, skin rash, itching, facial or lip swelling, joint pain, or swelling. There is no history of initiating new medications or alcohol intake, no known allergies or similar conditions, and no history of atopy. The patient did not have any previous history of ACEI-induced angioedema. Upon arrival, the patient was conscious with stable vital signs. Physical examination revealed a swollen uvula with normal tongue shape and movement (Fig. 1). Biphasic stridor was heard over the trachea on auscultation. No palpable masses or rashes were noted. A CT scan of the neck showed a pedunculated soft tissue mass measuring approximately 5 × 2 cm in the oropharynx, attached to the soft palate (Fig. 2 A: Sagittal view, B: Axial view). The main possibility considered was uvular angioedema.

The patient received emergency treatment with adrenaline, steroids, and antihistamines. His other chronic medications were stopped including dapagliflozin, tamsulosin, bisoprolol, aspirin, and atorvastatin. He was admitted to the intensive care unit (ICU) for observation and further management. An ENT specialist evaluated the patient by clinical evaluation and CT scan review and diagnosed him with Quincke's disease specifically isolated angioedema of the uvula, which was attributed to the use of Entresto (specifically, the valsartan component), other causes of Quincke's disease were excluded such as trauma or inhalation injury, and idiopathic. The patient was kept in the ICU for 24 hours and was hemodynamically stable. He was then transferred to the medical ward with continuous monitoring.

After three days of treatment (he was on Dexamethasone 4mg IV 1x1 and Famotidine 40 mg po 1x1), The patient's general condition improved significantly as the stridor subsided, and the patient tolerated oral intake well. A follow-up neck CT scan showed resolution of the previously observed findings, with no abnormalities detected during the examination.

After being discharged, the patient continued to take his prescribed medications, which included tamsulosin, aspirin, bisoprolol, atorvastatin, and dapagliflozin, without any problems. The physician's recommendation is to avoid ACEIs and ARB medications. The patient was started on hydralazine and isosorbide mononitrate medicine, however, Entresto was not resumed. After being discharged from the hospital, the patient was checked on at the internal medicine clinic one week later, he was in an improved and stable condition and had no issues.

## 3 Discussion

A nonpitting, asymmetrical swelling that is typically nonpruritic characterizes angioedema, which is a deep swelling of tissues under the skin and mucous membranes. It frequently affects the perioral region, periorbital region, tongue, genital region, and extremities [14]. Black race, female gender, previous drug rash [15], smoking habit [16], age over 65, seasonal allergies, recent initiation of ACEIs (first week of therapy), obesity, upper airway surgery or trauma, sleep apnea, and immunosuppression in cardiac and renal transplant recipients have all been identified as risk factors for ACEI-induced angioedema [1]. Moreover, according to Malde et al. [17], patients who are female and of African American descent are more likely to develop ACEI- or ARB-induced angioedema. Sondhi et al. [18] reported patients with ACEI-induced angioedema who were 64 % female and 91 % African-American, and they also observed similar outcomes.

Numerous cases of angioedema associated with ARBs are being reported as a result of the increased use of these medications [14,19]. in which cases, patients have been prescribed such drugs for more than 3 years. Moreover, several medications, including aspirin, non-steroidal anti-inflammatory drugs (NSAIDs), certain antibiotics, and angiotensin II receptor antagonists, can also cause this disease.

In our case Entresto was administrated for more than two years as well, indicating that the duration of medication is not related to the occurrence of angioedema. On the other hand, it's essential to consider if any previous ACEI-induced angioedema episode occurred with the patient or not. Several incidences of ACEIs and ARBs having a cross-reactive effect have been documented [[20], [21], [22], [23], [24]], which suggests that prior episodes caused by ACEIs can commonly be reported. Furthermore, since the patient is taking Entresto, sacubitril could contribute to causing angioedema since its effect of increasing bradykinin level is as same as ACEIs. A study evaluated the risk of angioedema among Sacubitril/valsartan new users compared with ACEIs and ARBS new users separately. As a result, compared to users of ACEIs or ARBs, they did not find that Sacubitril/valsartan new users had a higher risk of angioedema. However, compared to Sacubitril/valsartan new users, Sacubitril/valsartan users who recently moved from ACEIs or ARB had a higher incidence of angioedema [25]. On the other hand, a study reported that there was no discernible increase in the risk of angioedema comparing sacubitril/valsartan to enalapril [26].

As reported Angiotensin-converting enzyme (ACE) inhibitor use is frequently associated with the occurrence of drug-induced angioedema, which is likely to be an adverse effect of these medications' impact on bradykinin levels. If elevated levels of bradykinin are the main factor contributing to angioedema secondary to ACEI use, then ARBs are thought to cause very few, if any, occurrences of the condition. It is unknown how exactly these two kinds of medications cause angioedema. Several animal studies indicate a potential link between the use of ARBs and elevated tissue bradykinin levels as a result of activating the angiotensin II AT2 receptor [10]. ARBs, like ACEIs, work therapeutically by inhibiting the renin-angiotensin system. The AT1 receptor for angiogenic factor II is specifically blocked by ARBs. The competitive blockage of these AT1 receptors is assumed to be what causes their antihypertensive and cardiovascular effects. By preventing angiotensin II production, ACEIs regulate the renin-angiotensin system. These drugs do, however, prevent bradykinin from being broken down. In addition, vasodilation and vascular permeability have both been shown to be correlated with elevated bradykinin levels [19]. However, the role of AT2 receptors in cardiovascular hemodynamics is becoming more recognized. In contrast to I receptors, AT2 receptors are less affine for ARBs. At least a temporary rise in angiotensin II levels has been observed in humans when AT1 receptors are inhibited by ARBs. The expression and activation of AT2 receptors may be induced by rising angiotensin II levels. ARBs have been demonstrated to elevate renal and aortic bradykinin levels in animal models, an effect that is assumed to be attributable to elevated AT2 receptor stimulation. By inhibiting AT1 receptors and elevating angiotensin II levels, ARBs may exacerbate angioedema. The following increase in AT2 receptor activation caused by these elevated levels may result in elevated tissue bradykinin levels. Although the rise in bradykinin might not be enough to be observed in the serum, it might be high enough in the tissues to result in angioedema [[27], [28], [29], [30]]. Furthermore, another study [31] indicated that ARB treatment (50 mg losartan) increased the serum bradykinin level in hypertensive patients. According to this study, valsartan and other ARBs may cause angioedema by a similar mechanism as ACEIs. Due to the lack of such data and the exact mechanism of angioedema caused by ARBs is yet unknown, these explanations have limitations. More study is required to elucidate the pathophysiology of angioedema.

Despite the lack of information regarding the precise cause of ARB-induced angioedema and its lack of incidence, pharmacists, doctors, and other healthcare professionals should be aware that it can occur when using these medications. Additionally, while a history of angioedema brought on by an ACEI is not a strict prohibition against using an ARB, it should be taken into account when weighing the advantages and disadvantages of ARB therapy because there have been reports of cross-reactivity between ACEIs and ARBs [3].Also, neprilysin inhibitors should be considered when added to therapy regimens and clinicians should recognize the safety risk when prescribing sacubitril/valsartan and continue educating the patients about the symptoms and signs of angioedema [32].

## 4 Conclusion

Since Entresto medication is being used in clinical settings all over the world, practitioners and healthcare providers need to be aware of the pharmacologic and adverse effect profiles of these agents (sacubitril/valsartan) and educate the patients about the signs and symptoms of angioedema. This case and the relevant scientific literature are consistent with the hypothesis that ACEIs cause angioedema. As if the patient has similar negative symptoms with ACEI, the literature lists angioedema as a side effect of sacubitril/valsartan medication. However, even if the patient did not get angioedema after starting an ACEI, they could have it with sacubitril/valsartan.

## CRediT authorship contribution statement

**Maisa A. Nazzal:** Writing – review & editing, Writing – original draft, Validation, Supervision, Methodology, Investigation. **Abbas Iter:** Visualization, Methodology. **Ameed Q. Dawabsheh:** Writing – review & editing, Writing – original draft, Methodology. **Majd A. Bsharat:** Resources, Methodology, Data curation.

## Ethics approval and consent to participate

All participants/patients (or their proxies/legal guardians) provided informed consent for the publication of their anonymized case details and images. All aspects of the study protocols, including access to and use of the patient clinical information, were approved by Ibn Sina Specialized Hospital's Institutional Review Board (IRB). We fully explained all parts of the study to the patient and obtained their informed verbal consent before starting. The IRB of Ibn Sina Specialized Hospital approved verbal and written consent. The authors confirmed that all methods were performed following the relevant guidelines and regulations.

## Consent for publication

Written informed consent was obtained from the patient to publish this report in accordance with the journal's patient consent policy.

## Availability of data and material

Due to privacy, the datasets used and/or analyzed during the current study are available from the corresponding author on reasonable request.

## Data availability statement

The data associated with this study has not been deposited into a publicly available repository.

The data that has been used is confidential.

## Funding

No funding was received.

## Declaration of competing interest

The authors declare that they have no known competing financial interests or personal relationships that could have appeared to influence the work reported in this paper.

## Abbreviations

ACE: Angiotensin-converting enzyme
ACEI: Angiotensin-converting enzyme inhibitors
NSAID: Non -Steroidal Anti-inflammatory Drugs
ARBs: Angiotensin-receptor blockers
CABG: Coronary Artery Bypass Graft
LVEF: Left Ventricular Ejection Fraction
CT: Computed tomography
ENT: Ear, Nose and Throat
ICU: Intensive Care Unit
AT1: Angiotensin 1
AT2: Angiotensin 2
